# Screening and Interventions for Elder Mistreatment: Geriatric Emergency Department Guidelines 2.0 Systematic Review

**DOI:** 10.1111/acem.70373

**Published:** 2026-07-09

**Authors:** Daniel Baek, Satheesh Gunaga, Christian H. Nickel, Charles L. Maddow, Brianna Fitzgerald, Neha Bajaj, Shan W. Liu, Colin Eng Choon Ong, Kristin Lees Haggerty, Tony Rosen

**Affiliations:** ^1^ Department of Emergency Medicine Weill Cornell Medicine/NewYork‐Presbyterian Hospital New York New York USA; ^2^ Department of Emergency Medicine Henry Ford Health & Michigan State University Health Sciences Detroit Michigan USA; ^3^ Emergency Medicine Service Line Envision Healthcare Ann Arbor Michigan USA; ^4^ Department of Emergency Medicine University Hospital Basel, University of Basel Basel Switzerland; ^5^ Research Unit for Emergency Medicine Odense University Hospital Odense Denmark; ^6^ University of Texas Health Science Center at Houston Houston Texas USA; ^7^ School of Public Health University of Texas Houston Health Science Center Houston Texas USA; ^8^ Harvard Medical School Program in Global Primary Health Care Boston Massachusetts USA; ^9^ Department of Emergency Medicine Harvard Medical School/Massachusetts General Hospital Boston Massachusetts USA; ^10^ Department of Emergency Medicine Ng Teng Fong General Hospital, National University Health System Singapore Singapore; ^11^ Education Development Center, Inc. Waltham Massachusetts USA

## Abstract

**Background:**

Elder mistreatment is common, underreported, and associated with significant morbidity and mortality. Emergency department (ED) visits provide an important opportunity to identify elder mistreatment and initiate intervention, but the evidence for the impact of screening or intervention strategies in this clinical setting is not well characterized. Our goal was to conduct systematic literature reviews to describe the impact of ED‐based screening tools and ED‐initiated interventions for elder mistreatment.

**Methods:**

We conducted two systematic reviews following PRISMA 2020 guidelines using predefined Patient Intervention Comparator Outcome (PICO) frameworks. PICO 1 evaluated ED based screening tools for patients aged 60 years and older. PICO 2 evaluated ED initiated interventions for patients with known or suspected elder mistreatment. Searches were performed in MEDLINE, Embase, Web of Science, and Cochrane CENTRAL from December 2018 through December 2024 and supplemented by prior scoping reviews.

**Results:**

For PICO 1 (ED screening) the prior scoping review identified 5 studies and the updated search identified 208 additional articles, of which 5 studies underwent full‐text review. For PICO 2 (ED intervention), the prior scoping review identified 5 studies and the updated search identified 191 additional articles, of which 2 studies underwent full‐text review. No studies were identified that met full inclusion criteria for either PICO. Existing studies described multiple screening tools and multidisciplinary intervention models, but lacked comparator groups, standardized reference standards, or clinically meaningful outcome measures required for inclusion.

**Conclusions:**

These empty systematic reviews emphasize the large existing knowledge gaps about ED elder mistreatment screening and intervention. These gaps affect the ability to make practice recommendations and highlight opportunities for future rigorous research to inform guideline development.

## 1 Introduction

Elder mistreatment is common and associated with significant morbidity and mortality. This mistreatment, also frequently referred to as elder abuse, is defined as action or negligence against a vulnerable older adult that causes harm or risk of harm, either committed by a person in a relationship with an expectation of trust or when an older person is targeted based on age or disability [1, 2]. Elder mistreatment may include physical abuse, sexual abuse, caregiver neglect, financial exploitation, or verbal/psychological/emotional abuse, with many older adults experiencing multiple types of mistreatment concurrently or over time [3, 4]. As many as 15% of community‐dwelling older adults worldwide experience mistreatment each year [4, 5], with nursing home residents at even higher risk [6]. Mistreatment exposure can lead to profound medical consequences, including disability [7], depression [8], and three times greater one‐year mortality [9, 10, 11]. Many older adults endure elder mistreatment for years before having it discovered or dying, with only an estimated 1 in 24 cases reported to the authorities [12]. Much of the associated morbidity and mortality is likely due to this delay in identification and intervention [13]. Because of this, improving identification and intervention for elder mistreatment is a public health priority [14, 15]. As the geriatric population continues to dramatically rise due to increased longevity and population trends [16], the need to improve identification and intervention will continue to grow.

The emergency department (ED) is a uniquely positioned clinical setting for identifying elder mistreatment and initiating intervention. Older adults use the ED at higher rates than any other age group [17]. Further, many elder mistreatment victims have limited social interaction outside their caregivers, making healthcare encounters, including ED visits, important opportunities for clinicians to identify mistreatment. Existing research suggests that elder mistreatment victims have high overall healthcare utilization and may interact with multiple healthcare and community touchpoints, including outpatient primary care, emergency care, and hospital‐based services [18, 19, 20, 21]. Despite this broader healthcare engagement, ED utilization rates among mistreated older adults remain substantially elevated, with some studies reporting rates up to three times higher than those of non‐victims [18]. The prolonged and multidisciplinary nature of ED encounters further enhances the potential for identification [22, 23, 24]. Emergency Medical Services clinicians transporting many of these patients may also provide critical insights into home environments and potential safety concerns [22, 23, 24, 25]. EDs are also well‐positioned to initiate intervention for elder mistreatment when it is identified. ED clinicians are experienced in mandatory reporting and coordinating connections to social and community resources. In larger EDs and hospitals, consult services and ancillary professionals, including social workers and care managers/coordinators, may be available to lead or assist with intervention.

Despite the potential, formal identification, reporting, and structured response to elder mistreatment in the ED remain limited [26, 27]. Recognizing this gap, the original consensus–based Geriatric Emergency Department (GED) Guidelines, published in 2014 through collaboration between the Society of Academic Emergency Medicine, the American College of Emergency Physicians, the Emergency Nurses Association, and the American Geriatrics Society [28], highlighted the importance of ED protocols and education addressing elder mistreatment [29, 30]. However, these recommendations were not derived from a systematic or transparent evidence‐based process [31]. Notably, subsequent scoping reviews conducted through 2018 by Kayser and colleagues [32] and 2019 by Mercier and colleagues [33] found that, while ED‐based elder mistreatment screening and intervention were described in the literature, no studies evaluated the impact of screening or the comparative effectiveness of ED‐based interventions.

In response to significant advances in geriatric emergency medicine research and evolving clinical priorities since 2014, a large, multidisciplinary collaboration was established in 2020 to update the GED Guidelines [31] to ensure continued relevance, clinical utility, and evidence‐based rigor. This effort, termed GED Guidelines 2.0 [31], substantially changed the approach from the original, prioritizing methodological transparency, formalized evidence grading, and consensus building grounded in systematic reviews and meta‐analyses. Content‐specific working groups were convened to address high‐priority clinical domains, each conducting comprehensive evidence syntheses to inform guideline development [31]. The first essential step of this process was conducting a systematic review and meta‐analysis of existing literature [34, 35, 36, 37]. This manuscript presents the work of the elder mistreatment working group. To inform GED guideline development, our goal was to conduct updated systematic literature reviews and meta‐analyses to describe the impact of ED‐based screening tools and ED‐initiated interventions for elder mistreatment.

## 2 Methods

We approached our systematic searches for relevant literature in elder mistreatment screening and intervention conducted from November 29, 2018 through March 10, 2025, as updates of previously published structured scoping reviews of both screening and intervention (covering 1959—November 28, 2018) that used the same search strategy [32]. We planned to combine and synthesize results for each of screening and intervention from the scoping review and our literature search. Our search protocol was developed a priori and registered with the International Prospective Register of Systematic Reviews (PROSPERO; Registration No. CRD420251043893 and CRD420251054579). This study adheres to the Preferred Reporting Items for Systematic Reviews and Meta‐Analysis (PRISMA) [38]. These methods aligned with those undertaken for other topic areas within the GED Guidelines 2.0 update to ensure transparency and comparability [31].

### 2.1 Details of Previously Published Scoping Reviews

The methods of these previous scoping reviews, published by Kayser and colleagues in 2021, are described in detail elsewhere [32]. Briefly, the authors, including one member of the current research team (TR), developed two PICO questions, one exploring ED screening for elder mistreatment and one for intervention (included in Appendix S1). Using pre‐determined search strategies developed by a medical librarian for each PICO (search terms provided in Appendices S2 and S3), both searches were conducted using OVID Medline, EMBASE, CINAHL, Cochrane Central Register of Controlled Trials, and Applied Social Sciences Index from 1959 through November 28, 2018 [32]. The scoping review yielded 5 studies [39, 40, 41, 42, 43] describing ED screening and 5 studies [44, 45, 46, 47, 48] describing ED intervention [32]. Notably, none of these screening or intervention studies fulfilled all of the criteria in either PICO question. Despite these findings, the studies identified in the prior scoping reviews were reassessed because the finalized GED Guidelines 2.0 PICO frameworks included modest refinements to intervention and outcome definitions, and because reevaluation using a standardized systematic review methodology ensured consistent application of eligibility criteria across both previously identified and newly identified studies.

### 2.2 PICO Questions

Our research team assessed the PICO questions for screening and intervention used by Kayser and colleagues, discussed them in several meetings with the larger Geriatric ED Guidelines 2.0 group, and made minor changes [32]. The PICO questions used for the updated search and for this systematic review are shown in Tables 1 and 2. Compared with the previously used PICO 1, “targeted screening” was added to the intervention. Compared with the previously used PICO 2, “APS reporting” was removed from the intervention and comparison given that it may not represent usual care in many EDs. Together, these questions were designed to assess the effectiveness of ED‐based screening and intervention strategies, define the extent of existing comparative evidence available to support guideline development, and identify key evidence gaps requiring future investigation.

**TABLE 1:** Details of PICO 1.

Population: Emergency Department patients aged 60 years and older
Intervention: Universal or targeted screening for elder mistreatment (or elder abuse)
Comparison: Usual care (clinical identification based on EMS, nurse, and physician gestalt and standard practice)
Outcome: Improve the total number of cases identified, diagnostic accuracy, and long‐term safety outcomes, including potential harms, legal outcomes, functional outcomes, psychosocial outcomes, and healthcare utilization
Question: In emergency department patients aged 60 years and older, does universal or targeted screening for elder mistreatment (or elder abuse), compared to usual care (clinical identification based on EMS, nurse, and physician gestalt and standard practice), improve the total number of cases identified, diagnostic accuracy, and long‐term safety outcomes, including potential harms, legal outcomes, functional outcomes, psychosocial outcomes, and healthcare utilization?

**TABLE 2:** Details of PICO 2.

Population: Emergency Department patients aged 60 years who are previously known, newly found, or suspected victims of elder mistreatment (or elder abuse)
Intervention: ED‐based or ED‐initiated interventions
Comparison: Usual care
Outcomes: Improve short‐ and long‐term safety, health, legal, functional, and psychosocial outcomes
Question: In emergency department patients aged 60 years and older who are previously known, newly found, or suspected victims of elder mistreatment (or elder abuse), how do ED‐based or ED‐initiated interventions compare to usual care in improving short‐ and long‐term safety, health, legal, functional, and psychosocial outcomes?

### 2.3 Search Strategy

To update the reviews for screening and intervention conducted by Kayser and colleagues, the previously used search terms and strategy for each PICO were reviewed by the medical librarian on our team (MD), who determined that no changes were needed for either [32]. Therefore, we used the same search terms and strategy as Kayser and colleagues (Appendices S2 and S3) to search more recent literature [32]. Search strategies for both PICOs 1 and 2 used a combination of standardized terms and keywords, employing the controlled vocabulary of each database and plain language. For both searches, multiple variants of terms “aged,” “emergency department,” and “elder abuse” were used. For PICO 1, multiple variants of “screening” were added, and for PICO #2, variants of “victim,” “safety,” and “identified” in addition to “intervention.” We conducted both updated searches from November 28, 2018 to March 10, 2025 in OVID Medline, EMBASE, CINAHL, Cochrane Central Register of Controlled Trials, and Applied Social Sciences Index, consistent with the databases used in the prior reviews [32, 33].

### 2.4 Study Selection

We used the Covidence systematic review platform (Melbourne, Australia) to manage study selection for both reviews. Results obtained from the literature searches were de‐duplicated by author and imported into Covidence for screening by research team members. Initially, team members reviewed titles and abstracts to identify studies potentially eligible for inclusion and exclude those clearly ineligible. Full text articles of potentially eligible studies were then retrieved and assessed for inclusion. At the full text stage, all studies identified in the prior scoping reviews were also reassessed for eligibility [32].

We used the content of each PICO question to determine inclusion eligibility. For the title and abstract screening stage, the requirement for a comparison group was removed to maximize sensitivity and avoid exclusion of potentially relevant studies, but this requirement was applied during full text review. Each record at each stage was reviewed by two independent team members, with conflicts resolved by consensus at group meetings. We excluded abstracts, conference proceedings, narrative reviews, editorials, and commentaries. We limited inclusion to English language studies.

### 2.5 Data Extraction, Risk of Bias Assessment, and Data Synthesis (Planned but Not Performed)

We planned to extract data for included studies for both PICO questions, perform formal risk of bias assessments, and evaluate heterogeneity. These steps were not performed because no studies met inclusion criteria for either PICO question. However, as these processes were pre‐specified and will be necessary for future systematic reviews and meta‐analyses as evidence emerges, they are described in Appendix S4.

## 3 Results

**FIGURE 1:**
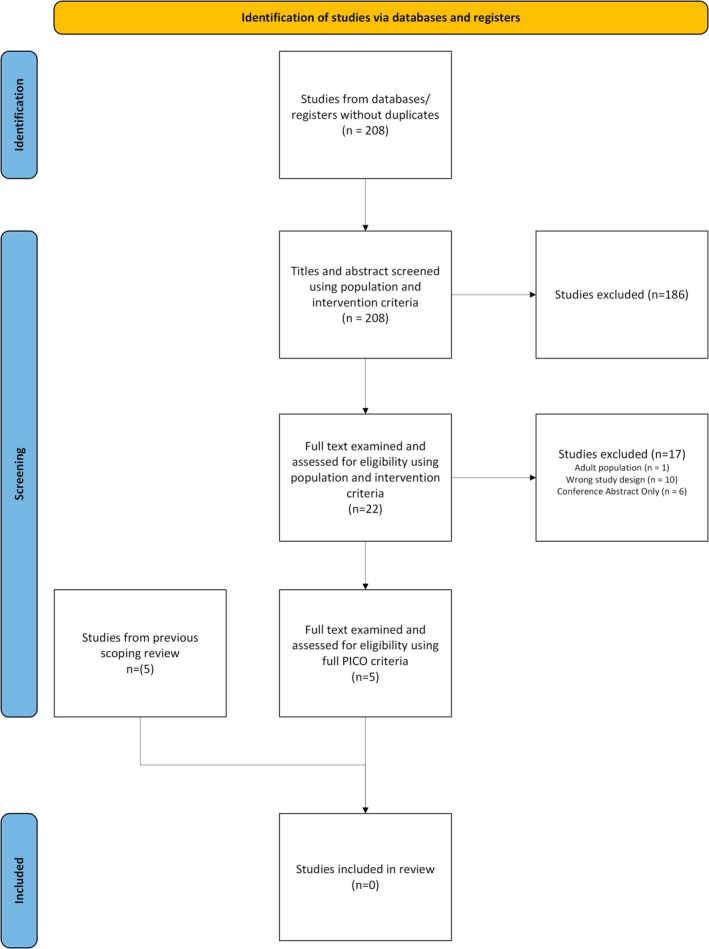
PRISMA Flow Chart for PICO 1.

**FIGURE 2:**
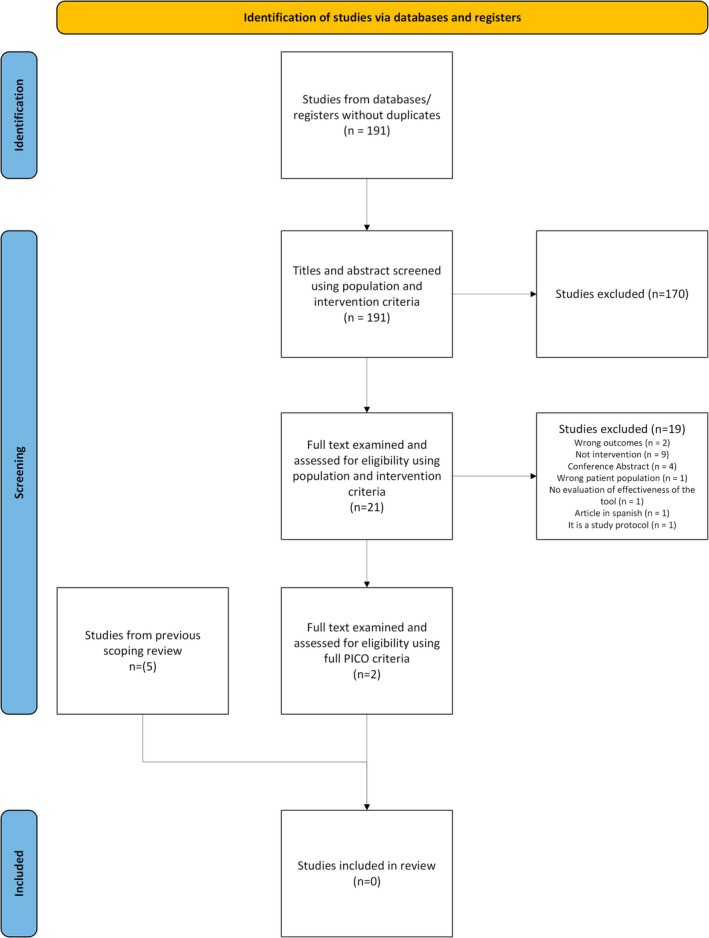
PRISMA Flow Chart for PICO 2.

The PRISMA diagrams detailing the number of studies identified and examined in the updated searches conducted for this systematic review are shown in Figures 1 and 2.

### 3.1 ED Screening for Elder Mistreatment (PICO 1)

For PICO 1 (ED screening), we identified 208 records. Following title and abstract screening, 5 studies underwent full‐text review, in addition to 5 studies identified from prior scoping review [32]. These studies met Population and Intervention criteria but did not meet full PICO inclusion criteria. Study characteristics and reasons for exclusion are summarized in Table 3.

**TABLE 3:** Emergency department‐based elder mistreatment screening studies: study characteristics, screening tools, and diagnostic performance.

Study	Source	Setting	*N*	Population	Design	Screening tool + reference standard	Key findings	Limitations (PICO relevance)
Fulmer (2000) [41]	Prior Scoping Review	1 US academic ED	36	≥ 70 years, caregiver ≥ 20 h/week, MMSE ≥ 18	Pilot validation	EAI; interdisciplinary NAT (geriatrician, SW, GNP)	Sens 71%, Spec 93%; 19.4% positive	No comparator; no outcome follow‐up
Fulmer (2005) [42]	Prior Scoping Review	4 US EDs	389	≥ 70 years, caregiver ≥ 20 h/week, MMSE ≥ 18	Diagnostic comparison	EAI; expert interdisciplinary team	Sens 24%, Spec 100%; 22.1% positive	No comparator; no outcome follow‐up
Eulitt (2014) [40]	Prior Scoping Review	2 US EDs (urban, rural)	180	≥ 65 years, stable, able to consent	Cross‐sectional	Modified ISAR	45.6% positive; higher risk in urban, low education, supervised settings	No comparator; no outcome follow‐up
Platts‐Mills (2018) [43]	Prior Scoping Review	1 US academic ED	259	≥ 65 years, non‐critical	Tool development	Candidate ED Senior AID items; assessor judgment	Sens 94%, Spec 90%; 6.6% suspected abuse	No comparator; no outcome follow‐up
Elman (2020) [39]	Prior Scoping Review	Expert panel	n/a	n/a	Delphi tool development	ED‐EMATS (social work tool)	First ED‐specific SW tool; 18 tools reviewed	No patient data
Platts‐Mills (2020) [49]	Updated Systematic Review	3 US academic EDs	916	≥ 65 years, non‐critical	Prospective diagnostic accuracy	ED Senior AID; LEAD panel	Sens 94.1%, Spec 84.3%; 1.9% positive; median time 87 s	No comparator; no outcome follow‐up
Richmond (2020) [50]	Updated Systematic Review	1 US academic ED	276	≥ 65 years, non‐critical	Observational	ED Senior AID + AMT4	High patient ability to self‐report (≥ 95% confident)	No reference standard; no comparator; no outcomes
Makaroun (2023) [51]	Updated Systematic Review	1 VA GED ED	251	≥ 65 years	QI pilot	EM‐SART	3.6% positive (screen), 2.0% confirmed; feasible workflow integration	No reference standard; no comparator; no outcomes
Riedel (2024) [52]	Updated Systematic Review	1 Swiss ED	1010	≥ 65 years	Prospective cohort	ED Senior AID (German)	2.9% positive; higher frailty, hospitalization; no mortality difference	No reference standard; no comparator
van Houten (2024) [53]	Updated Systematic Review	3 Dutch hospitals	386	≥ 70 years	Feasibility study	ERASE tool	3.9% positive; 88% clinician acceptability; 7.15 min completion	No reference standard; no comparator; no outcomes

*Note:* Studies met Population and Intervention criteria for PICO 1 but did not meet full inclusion criteria because no study included a comparator group or evaluated downstream patient centered outcomes attributable to screening.

Abbreviations: AMT4, Abbreviated Mental Test 4; APS, Adult Protective Services; EAI, Elder Assessment Instrument; ED, emergency department; ED‐EMATS, Emergency Department Elder Mistreatment Assessment Tool for Social Workers; ED Senior AID, Emergency Department Senior Abuse Identification; EM‐SART, Elder Mistreatment Screening and Response Tool; ERASE, EldeR AbuSE; ISAR, Identification of Seniors at Risk; LEAD, longitudinal, expert, all data; MMSE, Mini‐Mental Status Examination; NAT, Neglect Assessment Team; RA, research assistant; SW, social worker; US, United States; VA, Veterans Affairs.

Across included studies, six screening tools were described: the Elder Assessment Instrument (EAI) [41, 42], Identification of Seniors at Risk (ISAR) [40], Emergency Department Senior Abuse Identification (ED Senior AID) tool [43, 49], the Emergency Department Elder Mistreatment Assessment Tool for Social Workers (ED–EMATS), the Elder Mistreatment Screening and Response Tool (EM‐SART) [51], and the ERASE (EldeR AbuSE) [53]. Of these, test characteristics in comparison to a reference standard under research conditions were described for two (EAI [41, 42], ED Senior AID [43, 49]). Four studies described implementation or feasibility in clinical settings, including ISAR [40], ED Senior AID (German translation) [52], EM‐SART [51], and ERASE [53]. One study [52] examined longer‐term outcomes (mortality at 100 days) between patients screening positive and negative for elder mistreatment. No studies compared outcomes between screened and unscreened populations.

### 3.2 ED Intervention for Elder Mistreatment (PICO 2)

For PICO 2 (ED intervention), we identified 191 records. Following title and abstract screening, 2 studies underwent full‐text review, in addition to 5 studies identified from prior scoping review [32]. These studies met Population and Intervention criteria but did not meet full PICO inclusion criteria. Study characteristics and reasons for exclusion are summarized in Table 4.

**TABLE 4:** Emergency department‐based elder mistreatment intervention studies: study characteristics, intervention models, and key findings.

Study	Source	Setting	*N*	Population	Design	Intervention/model	Key findings	Limitations (PICO relevance)
Tomita (1982) [48]	Prior Scoping Review	1 US academic hospital	n/a	Not specified	Protocol	Interview guidance + suggested interventions	Conceptual protocol only	No patient data; no comparator
Carr (1986) [47]	Prior Scoping Review	1 US academic hospital	50	Hospitalized patients with suspected abuse	Protocol + case series	Referral system; multidisciplinary team (RN, MD, SW)	Team‐based approach identified suspected cases	No comparator; no outcome follow‐up
Jones (1988) [44]	Prior Scoping Review	1 US academic ED	36	Documented abuse/neglect	Retrospective review	ED protocol for identification and referral	Descriptive APS referral review	No comparator; no intervention evaluation
Matlaw (1994) [45]	Prior Scoping Review	1 US academic ED	130	≥ 60 years with suspected abuse	Observational (protocol‐based)	Multidisciplinary Elder Assessment Team (EAT)	69% confirmed abuse among suspected cases	No comparator; no outcome follow‐up
Rosen (2018) [46]	Prior Scoping Review	1 US academic ED	n/a	Suspected mistreatment	Protocol	Vulnerable Elder Protection Team (VEPT): SW + specialized provider, multidisciplinary coordination	Structured multidisciplinary intervention model	No patient data; no comparator
Rosen (2022) [54]	Updated Systematic Review	1 US academic ED	200	≥ 60 years, suspected mistreatment	Prospective cohort	VEPT consultation (multidisciplinary, APS, community linkage)	Consults increased (10 → 100/year); 62% high/mod suspicion; 75% discharge changes; strong provider acceptance	No comparator; limited outcome follow‐up
Baek (2025) [55]	Updated Systematic Review	2 US EDs (academic + community)	141	≥ 60 years, confirmed moderate/high suspicion	Prospective cohort (1‐year follow‐up)	VEPT + structured follow‐up (1, 6, 12 months)	60.9% mistreatment resolved at 12 months; reduced contact with perpetrator; 10.2% ED revisit with ongoing concern	No comparator

*Note:* Studies met Population and Intervention criteria for PICO 2 but did not meet full inclusion criteria because no study included a comparator group that did not receive the intervention.

Abbreviations: APS, Adult Protective Services; EAT, Elder Assessment Team; ED, emergency department; EMS, Emergency Medical Services; MD, physician; RN, registered nurse; SW, social worker; US, United States; VEPT, Vulnerable Elder Protection Team.

Two studies [44, 48] described structured assessment and response protocols, with one including clinical experience of referring cases to Adult Protective Services from the ED [44]. The remaining studies evaluated multidisciplinary ED‐ or hospital‐based intervention models, including the Multidisciplinary Elder Assessment Team [41] and the Vulnerable Elder Protection Team [46, 54, 55]. These studies reported on implementation characteristics, including consultation frequency, case identification, and multidisciplinary response processes.

A study evaluating a vulnerable elder protection team reported increases in consultation volume following program implementation and described associated care processes, including involvement of APS and coordination of social and community resources [54]. Studies on vulnerable elder protection teams also reported patient‐level outcomes following intervention, including changes in living situation and follow‐up assessments of ongoing mistreatment [54, 55]. No studies included a comparison group of patients who did not receive an intervention.

## 4 Discussion

These systematic reviews identified no studies meeting full PICO criteria for either ED screening or intervention strategies for elder mistreatment, resulting in two empty systematic reviews [56, 57]. This finding highlights a critical gap in the evidence base, as existing studies, while describing screening tools and intervention models, do not include comparative designs or patient‐centered outcomes necessary to evaluate effectiveness. As demonstrated in our results, prior work has focused largely on tool development, feasibility, and descriptive implementation, with limited validation against reference standards and no evaluation of outcomes comparing screened versus unscreened populations or intervention versus usual care. Compared with the prior GEAR scoping review, the updated literature reflects continued growth in operational, implementation‐focused, and multidisciplinary approaches to ED elder mistreatment care, despite the ongoing absence of comparative outcome‐based evidence. Collectively, these findings indicate that ED elder mistreatment research continues to expand in scope and clinical interest but has not yet advanced to the level of comparative outcome‐based evidence needed to meaningfully evaluate screening and intervention effectiveness.

The absence of studies meeting full inclusion criteria represents an important signal regarding the current state of the evidence to the research community. Empty systematic reviews are increasingly recognized as valuable markers of a field's developmental stage [56, 57]. They identify where evidence generation must occur before synthesis becomes meaningful. Within the GED Guidelines 2.0 initiative, the elder mistreatment working group intentionally retained comparative PICO frameworks focused on the most fundamental questions surrounding the impact of ED‐based screening and intervention strategies on patient outcomes. These questions represented the minimum evidentiary foundation necessary for future rigorous GRADE‐informed clinical practice guideline development. Although the group anticipated that direct comparative evidence might remain limited, the substantial growth of the elder mistreatment literature since the prior scoping review suggested that newer evaluative studies could exist. However, despite increasing publication activity in this area, the current literature remains largely limited to feasibility, implementation, and descriptive studies that cannot yet directly inform comparative effectiveness recommendations.

In the case of ED‐based screening and intervention for elder mistreatment, the absence of evidence meeting these PICO criteria reveals the need for stronger study design, prospective data collection, use of comparison subjects, and measurement of patient‐important outcomes. By applying a structured and reproducible methodology, these systematic reviews define a starting point from which future progress can be tracked. Future studies should be designed and conducted that would meet these criteria and therefore provide meaningful guidance to ED providers about how to optimally approach elder mistreatment screening and intervention, a high‐priority clinical issue.

Challenges in conducting research on elder mistreatment have been described previously and must be addressed to advance the field. These include (1) identifying and operationalizing an accurate and reproducible reference standard to assess for the presence or absence of mistreatment [58], (2) defining appropriate, patient‐centered outcomes that are likely to be impacted by screening and intervention and can be reliably measured [59], and (3) disaggregating this complex phenomenon into subtypes that may respond differently to specific screening and intervention strategies [60]. Additionally, the rigorous prospective studies needed to generate meaningful evidence will require substantial resources, necessitating prioritization by federal funding agencies and incentives for investigators to pursue this work. Pragmatic [61, 62] and stepped‐wedge [63, 64] study designs may offer feasible approaches in this context.

Implementation of ED‐based screening and intervention programs for elder mistreatment may also be challenging, even if supported by rigorous evidence. The ED is a hectic environment, and busy ED providers may initially be resistant to incorporating additional assessments or interventions into their workflow. Therefore, implementation science research [65, 66, 67] will be essential to optimize feasibility, integration, and sustained uptake in clinical practice. Further, screening and intervention for elder mistreatment are closely interrelated [32]. Screening programs that identify older adults being mistreated may highlight the urgent need for better response strategies [32]. Also, having proven, impactful mistreatment interventions may encourage providers to prioritize screening to find older adults who can benefit from them [32].

Given the multifactorial nature of elder mistreatment, future ED screening and intervention research should include multidisciplinary stakeholders, including ED physicians, nurses, technicians, social workers, inpatient hospital providers, Emergency Medical Services clinicians, Adult Protective Services, and law enforcement [23]. Interdisciplinary collaboration will be essential, and this should be guided and informed by the perspectives of older adults themselves [68], who are the intended beneficiaries of these efforts.

Importantly, the absence of current high‐quality evidence should be interpreted within the context of a rapidly evolving field. Several ongoing and recently initiated studies, including the Detection of Elder Abuse Through Emergency Care Technicians for Remote Primary Care (DETECT‐RPC) trial [69], the Raising the Identification of Self‐Neglect and Elder Abuse through Adult Protective Services (RISE‐APS) model [70], and emerging screening‐focused efforts such as the Mistreatment Screening in Elders Before Discharge (MISSED) trial [71, 72], are beginning to generate more rigorous data on the identification and management of elder mistreatment. In parallel, expansion of multidisciplinary response models such as the Vulnerable Elder Protection Team [54, 55] and implementation of the Geriatric Emergency Department Collaborative elder mistreatment toolkit [72] are contributing real‐world evidence on ED‐based workflows and outcomes. Collectively, these efforts suggest that a more robust evidence base may emerge in the near future to inform ED‐based screening and intervention strategies and support future guideline development.

## 5 Limitations

The systematic reviews we conducted were focused specifically on studies examining screening and intervention for elder mistreatment during emergency care. This represents only a small subset of the overall work in screening and intervention for elder mistreatment and does not include studies from other healthcare or community‐based settings. Such studies may offer insights about optimal approaches to address elder mistreatment in the ED. Though the original literature search [32] and our updated search were comprehensive, across multiple databases using librarian‐designed strategies and the study selection was handled rigorously, it is possible that relevant studies were not identified. Our approach also incorporated prior scoping reviews to capture earlier literature. This approach introduces the potential for discordance in comparison to a fully de novo systematic review across the entire time horizon. The search was limited to English language studies and extended through March 10, 2025, which may have excluded relevant non‐English studies [73] or more recently developed tools and interventions. Finally, heterogeneity in how elder mistreatment is defined and measured may limit the ability to capture all relevant work within a standardized systematic review framework.

## 6 Conclusion

We conducted systematic reviews to identify studies describing the impact of ED‐based screening tools and ED‐initiated interventions for elder mistreatment. No studies met full inclusion criteria for either review, as none included a comparator group that did not receive the screening or intervention. These findings highlight substantial gaps in the current evidence base, limiting the ability to inform ED‐specific clinical practice with direct, high‐quality evidence. While clinical practice guidelines can still be developed, they will necessarily rely on indirect evidence, data from outside emergency medicine, and lower certainty of evidence. Addressing these gaps will require rigorous, prospective studies with appropriate comparators, patient‐centered outcomes, and attention to implementation. Advancing this field will depend on coordinated, multidisciplinary efforts to generate evidence that can more directly guide ED‐based care for older adults at risk of mistreatment.

## Author Contributions

All authors made substantial contributions to the conception, design, and development of the protocol. All authors contributed to title and abstract review. D.B., S.G., C.H.N., C.L.M., S.W.L., T.R. contributed to full text review and data extraction. D.B., S.G., T.R. prepared the initial draft of the manuscript. C.H.N., C.L.M., B.F., N.B., S.W.L., C.E.C.O., K.L.H. revised the manuscript for important intellectual content. All authors approved the final version and agree both to be personally accountable for their contributions and to ensure that questions related to the accuracy or integrity of any part of the work, even ones in which they were not personally involved, are appropriately investigated, resolved, and the resolution documented in the literature.

## Funding

Funding was provided in part by The John A. Hartford foundation as part of the Geriatric Emergency Department Guidelines 2.0 effort. Support was also provided by the American Geriatrics Society. Kristin Lees Haggerty's participation was supported by K01 AG076992 from the National Institute on Aging. Tony Rosen's participation was supported by K24 AG084923 from the National Institute on Aging.

## Conflicts of Interest

Sateesh Gunaga, DO is a site Sub‐Investigator on an NIH‐funded study exploring ED dementia redesign (NIH Prime Award No. 1U19AG078105‐01A1; HFH Subaward No. 23‐A1‐00‐1007569). Dr. Gunaga serves as a volunteer member of the Board of Directors for Compassion & Choices, a 501(c) (3) nonprofit organization. Christian H. Nickel, MD is a member of the geriatric section of the EuropeanSociety of Emergency Medicine (EUSEM) and International Federation of Emergency Medicine (IFEM).

## Supporting information


**Appendix S1:** acem70373‐sup‐0001‐AppendicesS1‐S5.docx.

## Data Availability

Data sharing not applicable to this article as no datasets were generated or analysed during the current study.
