# Supplementary material for: Screening and Interventions for Elder Mistreatment: Geriatric Emergency Department Guidelines 2.0 Systematic Review
Source: Acad Emerg Med. 2026 Jul 9;33(7):e70373. doi: 10.1111/acem.70373 (PMC13348871; doi:10.1111/acem.70373)
Supplement: Supplementary file 1 — Appendix S1: acem70373‐sup‐0001‐AppendicesS1‐S5.docx. [file ACEM-33-0-s001.docx]

**SUPPLEMENTARY MATERIALS**

**Supplementary Material S1. PICO Question Details from Previous Scoping Review**

PICO #1

**Population:** Emergency department patients ≥ 60 years.

**Intervention:** Universal screening for elder abuse.

**Comparison:** 1. Targeted screening, 2. Usual care / Clinical identification of cases based on EMS, nurse, and physician gestalt and usual practice.

**Outcomes:** Total cases identified; accuracy of case identification; long-term safety outcomes including potential harms to the patient, short and long-term legal outcomes, short- and long-term functional outcomes, short- and long-term psychosocial outcomes, impact of screening on healthcare utilization.

**Question 1:** What is the effect of universal emergency department screening compared to targeted screening or usual practice on cases of elder abuse identified, accuracy of case identification, long-term safety outcomes including harms, legal, functional, and psychosocial outcomes, and health care utilization?

PICO #2

**Population:** Emergency department patients aged ≥ 60 years who are previously known, newly found, or suspected to be victims of elder abuse.

**Intervention:** Any emergency department-based or emergency department-initiated intervention including Adult Protective Services (APS) reporting.

**Comparison:** Usual care including APS reporting.

**Outcomes:** Short- and long-term safety, health, legal, functional, psychosocial outcomes

**Question 2:** What is the short and long-term safety, health, legal, functional, psychosocial impact of emergency department-based or emergency department-initiated interventions vs. usual care for emergency department patients previously known, newly found, or suspected to be victims of elder abuse?

**Supplementary Material S2. Literature Review Search Terms for PICO 1**

***PICO-1: AGED and Emergency department AND elder abuse AND screening***

Total: 301
Duplicates: 93
Unique: 208

ASSIA: 1
Central: 18
CINAHL: 47
Embase: 122
Ovid Medline: 113

**Ovid Medline**
113 results on 3/10/2025

Ovid MEDLINE(R) ALL <1946 to March 07, 2025>

1 exp Aged/ or (elderly or elderlies or centenarian* or nonagenarian* or octogenarian* or septuagenarian* or sexagenarian* or geriatric* or senium*).mp. or (senior* adj1 citizen*).mp. or ((older or frail*) adj2 (hospitalized or hospitalised or elder* or patient* or person* or inpatient* or people or adult*)).mp. or aged-adult*.mp. or aged-patient*.mp. or gerontolog*.jw. or geriatric*.jw. or ageing.jw. or aging.jw. or ((older or frail*) adj2 (hospitalized or hospitalised or elder* or patient* or person* or inpatient* or people or adult*)).mp. or 60 years old.mp. or (sixty adj4 "years old").mp. or 65 years old.mp. or (sixty-five adj4 "years old").mp. or ("Patients aged" adj3 "60 years").mp. or ("Patients aged" adj3 "65 years").mp. 3935294

2 exp Emergency Medical Services/ or exp hospital emergency service/ or emergicenter*.ti,ab. or (acute adj2 (setting or care or ward or hospital)).ti,ab. or (trauma adj1 (patient* or center* or centre* or department* or unit* or room*)).ti,ab. or ((Emergency or emergencies) adj3 (care or service* or dispatch* or department* or unit* or ward* or room* or center* or centre* or system* or stay* or admit* or admission* or evaluation* or assess* or medicine or visit* or Nurs* or physician* or patient* or clinician or personnel or accidents)).ti,ab. or ((EMS or ED or ER) adj3 (care or service* or dispatch* or department* or unit* or ward* or room* or center* or centre* or system* or stay* or admit* or admission* or evaluation* or assess* or visit* or Nurs* or physician* or patient* or personnel)).ti,ab. or (("a&e" or "a & e") adj2 (department* or admission* or admitted)).ti,ab. 421214

3 exp elder abuse/ or ((elder or elders or elderly or Older-adult* or aged-adult* or frail or geriatric* or senior*) adj3 (abuse* or neglect or maltreat* or mistreat* or exploit* or abandon*)).mp. or (physical* adj1 abuse*).mp. or ("family violence" or "domestic violence" or "domestic partner violence" or "domestic partner abuse" or "intimate partner violence").mp. 36335

4 exp risk assessment/ or (risk adj5 (assess* or abuse* or violen*)).mp. or screen*.mp. or assess*.mp. or identif*.mp. or evaluat*.mp. or scale*.mp. or consult*.mp. or interview*.mp. or surve*.mp. or questionnaire*.mp. or measure*.mp. or prescreen*.mp. or (injur* adj3 pattern*).mp. or "Identification of seniors at risk".mp. or "Triage risk screening tool".mp. or isar.mp. or trst.mp. 15347466

5 1 and 2 and 3 and 4 382

6 limit 5 to dt=20181128-20250310 113

**Embase.com**
122 results on 3/10/2025

1. 'aged'/exp OR elderly:ti,ab,kw,de OR elderlies:ti,ab,kw,de OR centenarian∗:ti,ab,kw,de OR nonagenarian∗:ti,ab,kw,de OR octogenarian∗:ti,ab,kw,de OR septuagenarian∗:ti,ab,kw,de OR sexagenarian∗:ti,ab,kw,de OR geriatric∗:ti,ab,kw,de OR senium∗:ti,ab,kw,de OR ((senior∗ NEAR/1 citizen∗):ti,ab,kw,de) OR (((older OR frail∗) NEAR/2 (hospitalized OR hospitalised OR elder∗ OR patient∗ OR person∗ OR inpatient∗ OR people OR adult∗)):ti,ab,kw,de) OR 'aged adult':ti,ab,kw,de OR 'aged adults':ti,ab,kw,de OR 'aged patient':ti,ab,kw,de OR 'aged patients':ti,ab,kw,de OR gerontolog∗:jt OR geriatric∗:jt OR ageing:jt OR aging:jt

2. 'emergency health service'/exp OR 'emergency ward'/exp OR emergicenter∗:ti,ab,kw,de OR ((acute NEAR/2 (setting OR care OR ward OR hospital)):ti,ab) OR ((trauma NEAR/1 (patient∗ OR center∗ OR centre∗ OR department∗ OR unit∗ OR room∗)):ti,ab) OR (((emergency OR emergencies) NEAR/3 (care OR service∗ OR dispatch∗ OR department∗ OR unit∗ OR ward∗ OR room∗ OR center∗ OR centre∗ OR system∗ OR stay∗ OR admit∗ OR admission∗ OR evaluation∗ OR assess∗ OR medicine OR visit∗ OR nurs∗ OR physician∗ OR clinician∗ OR personnel OR patient∗ OR accidents)):ti,ab,kw,de) OR (((ems OR ed OR er) NEAR/3 (care OR service∗ OR dispatch∗ OR department∗ OR unit∗ OR ward∗ OR room∗ OR center∗ OR centre∗ OR system∗ OR stay∗ OR admit∗ OR admission∗ OR evaluation∗ OR assess∗ OR visit∗ OR nurs∗ OR physician∗ OR patient∗ OR personnel)):ti,ab,kw,de) OR ((('a&e' OR 'a & e') NEAR/2 (department∗ OR admission∗ OR admitted)):ti,ab)

3. ‘elder abuse’/exp OR ‘elderly abuse’/exp OR ((elder or aged or elderly OR ‘Older adult’ OR ‘older adults’ OR ‘aged adults’ OR ‘aged adult’ OR senior* or geriatric*) near/3 (abuse* OR neglect* OR maltreat* OR mistreat* OR exploit* OR abandon*)):ti,ab,kw OR ((elder* OR "Older adult" OR "older adults" OR "aged adult" OR "aged adults" OR senior* OR geriatric*):ti,ab,kw AND ((physical near/1 abuse):ti,ab,kw OR family-violence:ti,ab,kw OR (domestic near/2 (violence OR abuse*)):ti,ab,kw OR intimate-partner-violence:ti,ab,kw OR intimate-partner-abuse:ti,ab,kw))

4. 'risk assessment'/exp OR ((risk NEAR/3 assess*):ti,ab,kw,de) OR screen*:ti,ab,kw,de OR assess*:ti,ab,kw,de OR identif*:ti,ab,kw,de OR interview*:ti,ab,kw,de OR scale*:ti,ab,kw,de OR evaluat*:ti,ab,kw,de OR consult*:ti,ab,kw,de OR surve*:ti,ab,kw,de OR questionnaire*:ti,ab,kw,de OR measure*:ti,ab,kw,de OR prescreen*:ti,ab,kw,de OR 'identification of seniors at risk':ti,ab,kw,de OR 'triage risk screening tool':ti,ab,kw,de OR isar:ti,ab,kw,de OR trst:ti,ab,kw,de

5. #1 AND #2 AND #3 AND #4

6. #5 AND [28-11-2018]/sd NOT [11-03-2025]/sd

**CINAHL Plus**
47 results 3/10/2025

1. MW “Aged” OR AB (elder OR elders OR elderly OR elderlies OR centenarian OR nonagenarian OR octogenarian OR septuagenarian OR sexagenarian OR geriatric* OR senium*) OR AB (sixty N4 ("years old")) OR AB (senior* N1 citizen*) OR AB ((older OR frail*) N2 (hospitalized OR hospitalised OR elder* OR patient* OR person* OR inpatient* OR people OR adult*)) OR AB “aged adult” OR AB “aged adults” OR AB “aged patient” OR AB “aged patients” OR AB ("Patients aged" N3 "60 years") OR AB ("Patients aged" N3 "65 years") Or TI (elder OR elders OR elderly OR elderlies OR centenarian OR nonagenarian OR octogenarian OR septuagenarian OR sexagenarian OR geriatric* OR senium*) OR TI (sixty N4 ("years old")) OR TI (senior* N1 citizen*) OR TI ((older OR frail*) N2 (hospitalized OR hospitalised OR elder* OR patient* OR person* OR inpatient* OR people OR adult*)) OR TI “aged adult” OR TI “aged adults” OR TI “aged patient” OR TI “aged patients” OR TI ("Patients aged" N3 "60 years") OR TI ("Patients aged" N3 "65 years") OR (SO gerontolog∗ OR geriatric∗ OR ageing OR aging)

2. MW “Emergency Medical Services” OR MW “hospital emergency service” OR AB emergicenter* OR AB Triage* OR AB (trauma N1 support) OR AB ((Emergency OR emergencies OR EMS OR ED) N2 (care OR service* OR dispatch* OR department* OR unit* OR ward* OR room* OR center* OR centre* OR system*)) OR TI emergicenter* OR TI Triage* OR TI (trauma N1 (support OR center OR centre OR department* OR unit* OR room*)) OR TI ((Emergency OR emergencies OR EMS OR ED) N2 (care OR service* OR dispatch* OR department* OR unit* OR ward* OR room* OR center* OR centre* OR system*))

3. MW “elder abuse” OR AB ((elder or elders OR aged or elderly OR "Older adult" OR "older adults" OR senior* OR geriatric*) N3 (abuse* OR neglect OR maltreatment OR mistreatment OR exploitation OR abandonment)) OR TI ((elder or aged or elderly OR "Older adult" OR "older adults" OR physical OR senior) N3 (abuse* OR neglect OR maltreatment OR mistreatment OR exploitation OR abandonment)) OR TI ((elder* OR "Older adult" OR "older adults" OR "aged adult" OR "aged adults" OR senior* OR geriatric*) N3 ("family violence" OR "domestic violence" OR "domestic partner abuse" OR "intimate partner violence")) OR AB ((elder* OR "Older adult" OR "older adults" OR "aged adult" OR "aged adults" OR senior* OR geriatric*) N3 ("family violence" OR "domestic violence" OR "domestic partner abuse" OR "intimate partner violence"))

4. MW “risk assessment” OR AB (risk* N3 assess*) OR AB screen* OR AB assess* OR AB identif* OR AB evaluat* OR AB consult* OR AB Surve* OR AB questionnaire* OR AB measure* OR AB “pre screening” OR AB "Identification of seniors at risk" OR AB "Triage risk screening tool" OR AB isar OR AB trst OR TI interview* OR AB interview* OR TI scale* OR AB scale* OR TI (risk* N3 assess*) OR TI screen* OR TI assess* OR TI identif* OR TI evaluat* OR TI consult* OR TI Surve* OR TI questionnaire* OR TI measure* OR TI “pre screening” OR TI "Identification of seniors at risk" OR TI "Triage risk screening tool" OR TI isar OR TI trst

5. #1 AND #2 AND #3 AND #4 AND Limiters - Publication Date: 20181101-20250331

**Cochrane Central**18 results on 3/10/2025

1. elderly:ti,ab,kw OR elderlies:ti,ab,kw OR centenarian*:ti,ab,kw OR nonagenarian*:ti,ab,kw OR octogenarian*:ti,ab,kw OR septuagenarian*:ti,ab,kw OR sexagenarian*:ti,ab,kw OR geriatric*:ti,ab,kw OR senium*:ti,ab,kw OR ((senior* NEAR/1 citizen*):ti,ab,kw ) OR (((older OR frail*) NEAR/2 (hospitalized OR hospitalised OR elder* OR patient* OR person* OR inpatient* OR people OR adult*)):ti,ab,kw ) OR 'aged adult':ti,ab,kw OR 'aged adults':ti,ab,kw OR 'aged patient':ti,ab,kw OR 'aged patients':ti,ab,kw

2. emergicenter*:ti,ab,kw OR ((acute NEAR/2 (setting OR care OR ward OR hospital)):ti,ab) OR ((trauma NEAR/1 (patient* OR center* OR centre* OR department* OR unit* OR room*)):ti,ab) OR (((emergency OR emergencies) NEAR/3 (care OR service* OR dispatch* OR department* OR unit* OR ward* OR room* OR center* OR centre* OR system* OR stay* OR admit* OR admission* OR evaluation* OR assess* OR medicine OR visit* OR nurs* OR physician* OR clinician* OR personnel OR patient* OR accidents)):ti,ab,kw ) OR (((ems OR ed OR er) NEAR/3 (care OR service* OR dispatch* OR department* OR unit* OR ward* OR room* OR center* OR centre* OR system* OR stay* OR admit* OR admission* OR evaluation* OR assess* OR visit* OR nurs* OR physician* OR patient* OR personnel)):ti,ab,kw ) OR ((('a&e' OR 'a & e') NEAR/2 (department* OR admission* OR admitted)):ti,ab)

3. ((elder or aged or elderly OR ‘Older adult’ OR ‘older adults’ OR ‘aged adults’ OR ‘aged adult’ OR senior* or geriatric*) near/3 (abuse* OR neglect* OR maltreat* OR mistreat* OR exploit* OR abandon*)):ti,ab,kw OR ((elder* OR "Older adult" OR "older adults" OR "aged adult" OR "aged adults" OR senior* OR geriatric*):ti,ab,kw AND ((physical near/1 abuse):ti,ab,kw OR family-violence:ti,ab,kw OR (domestic near/2 (violence OR abuse*)):ti,ab,kw OR intimate-partner-violence:ti,ab,kw OR intimate-partner-abuse:ti,ab,kw))

4. ((risk NEAR/3 assess*):ti,ab,kw ) OR screen*:ti,ab,kw OR assess*:ti,ab,kw OR identif*:ti,ab,kw OR interview*:ti,ab,kw OR scale*:ti,ab,kw OR evaluat*:ti,ab,kw OR consult*:ti,ab,kw OR surve*:ti,ab,kw OR questionnaire*:ti,ab,kw OR measure*:ti,ab,kw OR prescreen*:ti,ab,kw OR 'identification of seniors at risk':ti,ab,kw OR 'triage risk screening tool':ti,ab,kw OR isar:ti,ab,kw OR trst:ti,ab,kw

5. #1 AND #2 AND #3 AND #4

6. #5 AND Date added to CENTRAL trials database Custom Range: 28/11/2018 to 10/03/2025

**Applied Social Sciences Index and Abstracts (ASSIA**1 result on 3/10/2025 with **Applied filters:** 2018-11-28 - 2025-03-10

noft("elder abuse") AND noft("emergency medical services" OR "emergency service" OR "emergency service" OR "emergency department" OR "emergency room" OR "Emergency center" OR "trauma center" )

**Supplementary Material S3. Literature Review Search Terms for PICO 2**

PICO-2: AGED and Emergency department AND elder abuse AND victims

Total: 275
Duplicates: 84
Unique: 191

ASSIA: 1
Cochrane Central: 12
CINAHL: 45
Embase: 103
Ovid Medline: 114

Ovid Medline

Ovid MEDLINE(R) ALL <1946 to March 07, 2025>

1 exp Aged/ or (elderly or elderlies or centenarian* or nonagenarian* or octogenarian* or septuagenarian* or sexagenarian* or geriatric* or senium*).mp. or (senior* adj1 citizen*).mp. or ((older or frail*) adj2 (hospitalized or hospitalised or elder* or patient* or person* or inpatient* or people or adult*)).mp. or aged-adult*.mp. or aged-patient*.mp. or gerontolog*.jw. or geriatric*.jw. or ageing.jw. or aging.jw. or ((older or frail*) adj2 (hospitalized or hospitalised or elder* or patient* or person* or inpatient* or people or adult*)).mp. or 60 years old.mp. or (sixty adj4 "years old").mp. or 65 years old.mp. or (sixty-five adj4 "years old").mp. or ("Patients aged" adj3 "60 years").mp. or ("Patients aged" adj3 "65 years").mp. 3935294

2 exp Emergency Medical Services/ or exp hospital emergency service/ or emergicenter*.ti,ab. or (acute adj2 (setting or care or ward or hospital)).ti,ab. or (trauma adj1 (patient* or center* or centre* or department* or unit* or room*)).ti,ab. or ((Emergency or emergencies) adj3 (care or service* or dispatch* or department* or unit* or ward* or room* or center* or centre* or system* or stay* or admit* or admission* or evaluation* or assess* or medicine or visit* or Nurs* or physician* or patient* or clinician or personnel or accidents)).ti,ab. or ((EMS or ED or ER) adj3 (care or service* or dispatch* or department* or unit* or ward* or room* or center* or centre* or system* or stay* or admit* or admission* or evaluation* or assess* or visit* or Nurs* or physician* or patient* or personnel)).ti,ab. or (("a&e" or "a & e") adj2 (department* or admission* or admitted)).ti,ab. 421214

3 exp elder abuse/ or ((elder or elders or elderly or Older-adult* or aged-adult* or frail or geriatric* or senior*) adj3 (abuse* or neglect or maltreat* or mistreat* or exploit* or abandon*)).mp. or (physical* adj1 abuse*).mp. or ("family violence" or "domestic violence" or "domestic partner violence" or "domestic partner abuse" or "intimate partner violence").mp. 36335

4 Elder Abuse/di or Elder Abuse/th or Elder Abuse/rh or victim*.mp. or identif*.mp. or known.mp. or suspicio*.mp. or "protective services".mp. or injur*.mp. or safety.mp. or unsafe.mp. or case*.mp. or (medical adj1 marker*).mp. 12437783

5 1 and 2 and 3 and 4 388

6 limit 5 to dt=20181128-20250310 114

Embase.com
103 results on 3/10/2025

1. 'aged'/exp OR elderly:ti,ab,kw,de OR elderlies:ti,ab,kw,de OR centenarian∗:ti,ab,kw,de OR nonagenarian∗:ti,ab,kw,de OR octogenarian∗:ti,ab,kw,de OR septuagenarian∗:ti,ab,kw,de OR sexagenarian∗:ti,ab,kw,de OR geriatric∗:ti,ab,kw,de OR senium∗:ti,ab,kw,de OR ((senior∗ NEAR/1 citizen∗):ti,ab,kw,de) OR (((older OR frail∗) NEAR/2 (hospitalized OR hospitalised OR elder∗ OR patient∗ OR person∗ OR inpatient∗ OR people OR adult∗)):ti,ab,kw,de) OR 'aged adult':ti,ab,kw,de OR 'aged adults':ti,ab,kw,de OR 'aged patient':ti,ab,kw,de OR 'aged patients':ti,ab,kw,de OR gerontolog∗:jt OR geriatric∗:jt OR ageing:jt OR aging:jt

2. 'emergency health service'/exp OR 'emergency ward'/exp OR emergicenter∗:ti,ab,kw,de OR ((acute NEAR/2 (setting OR care OR ward OR hospital)):ti,ab) OR ((trauma NEAR/1 (patient∗ OR center∗ OR centre∗ OR department∗ OR unit∗ OR room∗)):ti,ab) OR (((emergency OR emergencies) NEAR/3 (care OR service∗ OR dispatch∗ OR department∗ OR unit∗ OR ward∗ OR room∗ OR center∗ OR centre∗ OR system∗ OR stay∗ OR admit∗ OR admission∗ OR evaluation∗ OR assess∗ OR medicine OR visit∗ OR nurs∗ OR physician∗ OR clinician∗ OR personnel OR patient∗ OR accidents)):ti,ab,kw,de) OR (((ems OR ed OR er) NEAR/3 (care OR service∗ OR dispatch∗ OR department∗ OR unit∗ OR ward∗ OR room∗ OR center∗ OR centre∗ OR system∗ OR stay∗ OR admit∗ OR admission∗ OR evaluation∗ OR assess∗ OR visit∗ OR nurs∗ OR physician∗ OR patient∗ OR personnel)):ti,ab,kw,de) OR ((('a&e' OR 'a & e') NEAR/2 (department∗ OR admission∗ OR admitted)):ti,ab)

3. ‘elder abuse’/exp OR ‘elderly abuse’/exp OR ((elder or aged or elderly OR ‘Older adult’ OR ‘older adults’ OR ‘aged adults’ OR ‘aged adult’ OR senior* or geriatric*) near/3 (abuse* OR neglect* OR maltreat* OR mistreat* OR exploit* OR abandon*)):ti,ab,kw OR ((elder* OR "Older adult" OR "older adults" OR "aged adult" OR "aged adults" OR senior* OR geriatric*):ti,ab,kw AND ((physical near/1 abuse):ti,ab,kw OR family-violence:ti,ab,kw OR (domestic near/2 (violence OR abuse*)):ti,ab,kw OR intimate-partner-violence:ti,ab,kw OR intimate-partner-abuse:ti,ab,kw))

4. 'victim'/de OR victim*:ti,ab,kw OR identif*:ti,ab,kw,de OR known:ti,ab,kw,de OR ((abuse NEAR/3 (diagnos* OR determine* OR detect* OR treat* OR rehabilita* OR protect*)):ti,ab,kw) OR suspicio*:ti,ab,kw,de OR 'protective services':ti,ab,kw,de OR safety:ti,ab,kw,de OR unsafe:ti,ab,kw,de OR case*:ti,ab,kw,de OR ((medical NEAR/1 marker*):ti,ab,kw,de) OR suffer*:ti,ab,kw,de

5. #1 AND #2 AND #3 AND #4

6. #5 AND [28-11-2018]/sd NOT [11-03-2025]/sd

**CINAHL Plus**
47 results 3/10/2025

1. MW “Aged” OR AB (elder OR elders OR elderly OR elderlies OR centenarian OR nonagenarian OR octogenarian OR septuagenarian OR sexagenarian OR geriatric* OR senium*) OR AB (sixty N4 ("years old")) OR AB (senior* N1 citizen*) OR AB ((older OR frail*) N2 (hospitalized OR hospitalised OR elder* OR patient* OR person* OR inpatient* OR people OR adult*)) OR AB “aged adult” OR AB “aged adults” OR AB “aged patient” OR AB “aged patients” OR AB ("Patients aged" N3 "60 years") OR AB ("Patients aged" N3 "65 years") Or TI (elder OR elders OR elderly OR elderlies OR centenarian OR nonagenarian OR octogenarian OR septuagenarian OR sexagenarian OR geriatric* OR senium*) OR TI (sixty N4 ("years old")) OR TI (senior* N1 citizen*) OR TI ((older OR frail*) N2 (hospitalized OR hospitalised OR elder* OR patient* OR person* OR inpatient* OR people OR adult*)) OR TI “aged adult” OR TI “aged adults” OR TI “aged patient” OR TI “aged patients” OR TI ("Patients aged" N3 "60 years") OR TI ("Patients aged" N3 "65 years") OR (SO gerontolog∗ OR geriatric∗ OR ageing OR aging)

2. MW “Emergency Medical Services” OR MW “hospital emergency service” OR AB emergicenter* OR AB Triage* OR AB (trauma N1 support) OR AB ((Emergency OR emergencies OR EMS OR ED) N2 (care OR service* OR dispatch* OR department* OR unit* OR ward* OR room* OR center* OR centre* OR system*)) OR TI emergicenter* OR TI Triage* OR TI (trauma N1 (support OR center OR centre OR department* OR unit* OR room*)) OR TI ((Emergency OR emergencies OR EMS OR ED) N2 (care OR service* OR dispatch* OR department* OR unit* OR ward* OR room* OR center* OR centre* OR system*))

3. MW “elder abuse” OR AB ((elder or elders OR aged or elderly OR "Older adult" OR "older adults" OR senior* OR geriatric*) N3 (abuse* OR neglect OR maltreatment OR mistreatment OR exploitation OR abandonment)) OR TI ((elder or aged or elderly OR "Older adult" OR "older adults" OR physical OR senior) N3 (abuse* OR neglect OR maltreatment OR mistreatment OR exploitation OR abandonment)) OR TI ((elder* OR "Older adult" OR "older adults" OR "aged adult" OR "aged adults" OR senior* OR geriatric*) N3 ("family violence" OR "domestic violence" OR "domestic partner abuse" OR "intimate partner violence")) OR AB ((elder* OR "Older adult" OR "older adults" OR "aged adult" OR "aged adults" OR senior* OR geriatric*) N3 ("family violence" OR "domestic violence" OR "domestic partner abuse" OR "intimate partner violence"))

4. TI victim* OR TI identif* OR TI known OR (MH "Elder Abuse/DI/TH/RH") OR (MH fractures) OR (MH “wounds and injuries”) OR TI suspicio* OR TI suspect* OR TI "protective services" OR TI injur* OR TI safety OR TI unsafe OR TI case* OR TI (medical N1 marker*) OR AB victim* OR AB identif* OR AB known OR AB suspicio* OR AB suspect* OR AB "protective services" OR AB injur* OR AB safety OR AB unsafe OR AB case* OR AB (medical N1 marker*)

5. #1 AND #2 AND #3 AND #4 AND Limiters - Publication Date: 20181101-20250331

**Applied Social Sciences Index and Abstracts (ASSIA)**1 result on 3/10/2025 with **Applied filters:** 2018-11-28 - 2025-03-10

noft("elder abuse") AND noft("emergency medical services" OR "emergency service" OR "emergency service" OR "emergency department" OR "emergency room" OR "Emergency center" OR "trauma center" )

**Cochrane Central**12 results on 3/10/2025

1. elderly:ti,ab,kw OR elderlies:ti,ab,kw OR centenarian*:ti,ab,kw OR nonagenarian*:ti,ab,kw OR octogenarian*:ti,ab,kw OR septuagenarian*:ti,ab,kw OR sexagenarian*:ti,ab,kw OR geriatric*:ti,ab,kw OR senium*:ti,ab,kw OR ((senior* NEAR/1 citizen*):ti,ab,kw ) OR (((older OR frail*) NEAR/2 (hospitalized OR hospitalised OR elder* OR patient* OR person* OR inpatient* OR people OR adult*)):ti,ab,kw ) OR 'aged adult':ti,ab,kw OR 'aged adults':ti,ab,kw OR 'aged patient':ti,ab,kw OR 'aged patients':ti,ab,kw

2. emergicenter*:ti,ab,kw OR ((acute NEAR/2 (setting OR care OR ward OR hospital)):ti,ab) OR ((trauma NEAR/1 (patient* OR center* OR centre* OR department* OR unit* OR room*)):ti,ab) OR (((emergency OR emergencies) NEAR/3 (care OR service* OR dispatch* OR department* OR unit* OR ward* OR room* OR center* OR centre* OR system* OR stay* OR admit* OR admission* OR evaluation* OR assess* OR medicine OR visit* OR nurs* OR physician* OR clinician* OR personnel OR patient* OR accidents)):ti,ab,kw ) OR (((ems OR ed OR er) NEAR/3 (care OR service* OR dispatch* OR department* OR unit* OR ward* OR room* OR center* OR centre* OR system* OR stay* OR admit* OR admission* OR evaluation* OR assess* OR visit* OR nurs* OR physician* OR patient* OR personnel)):ti,ab,kw ) OR ((('a&e' OR 'a & e') NEAR/2 (department* OR admission* OR admitted)):ti,ab)

3. ((elder or aged or elderly OR ‘Older adult’ OR ‘older adults’ OR ‘aged adults’ OR ‘aged adult’ OR senior* or geriatric*) near/3 (abuse* OR neglect* OR maltreat* OR mistreat* OR exploit* OR abandon*)):ti,ab,kw OR ((elder* OR "Older adult" OR "older adults" OR "aged adult" OR "aged adults" OR senior* OR geriatric*):ti,ab,kw AND ((physical near/1 abuse):ti,ab,kw OR family-violence:ti,ab,kw OR (domestic near/2 (violence OR abuse*)):ti,ab,kw OR intimate-partner-violence:ti,ab,kw OR intimate-partner-abuse:ti,ab,kw))

4. victim*:ti,ab,kw OR identif*:ti,ab,kw OR known:ti,ab,kw OR ((abuse NEAR/3 (diagnos* OR determine* OR detect* OR treat* OR rehabilita* OR protect*)):ti,ab,kw) OR suspicio*:ti,ab,kw OR 'protective services':ti,ab,kw OR safety:ti,ab,kw OR unsafe:ti,ab,kw OR case*:ti,ab,kw OR ((medical NEAR/1 marker*):ti,ab,kw ) OR suffer*:ti,ab,kw

5. #1 AND #2 AND #3 AND #4

6. #5 AND Date added to CENTRAL trials database Custom Range: 28/11/2018 to 10/03/2025

**Supplementary Material S4: Data Extraction, Risk of Bias Assessment, Synthesis of Evidence (Planned but Not Performed)**

We describe here in detail the planned but not performed activities for this systematic review and meta-analysis. We believe that doing so may be helpful, given that we planned these activities and that they will be necessary for rigorous future reviews and meta-analyses that update our findings as evidence becomes available.

*Data Extraction (Planned but Not Performed)*

We planned to develop, pilot, and finalize a structured form for data abstraction. We anticipated collecting study characteristics, population details, intervention and comparator definitions, tool domains, screener type, reference standards, and outcomes. Two reviewers were to extract data independently and reconcile discrepancies by consensus.

*Risk of Bias Assessment (Planned but Not Performed)*

We pre-specified design-specific tools: Cochrane RoB 2^66^ for randomized trials, ROBINS-I^67^ for non-randomized studies, and QUADAS-2^68^ for diagnostic accuracy studies. We planned to rate each domain as low, high, or unclear risk.

*Data Synthesis and Certainty of Evidence (Planned but Not Performed)*

We planned to conduct quantitative synthesis using random-effects meta-analysis for comparable outcomes and hierarchical summary receiver-operating-characteristic (HSROC) modeling for diagnostic accuracy data. Heterogeneity was to be assessed with the I² statistic and χ² test, with planned sensitivity and subgroup analyses by screening strategy, tool type, screener, and setting.


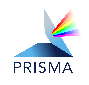
**Supplementary Material S5: PRISMA 2020 Checklist**

| **Section and Topic** | **Item #** | **Checklist item** | **Location where item is reported** |
| --- | --- | --- | --- |
| **TITLE** | | |  |
| Title | 1 | Identify the report as a systematic review. | 1 |
| **ABSTRACT** | | |  |
| Abstract | 2 | See the PRISMA 2020 for Abstracts checklist. | 2 |
| **INTRODUCTION** | | |  |
| Rationale | 3 | Describe the rationale for the review in the context of existing knowledge. | 3-5 |
| Objectives | 4 | Provide an explicit statement of the objective(s) or question(s) the review addresses. | 3-5 |
| **METHODS** | | |  |
| Eligibility criteria | 5 | Specify the inclusion and exclusion criteria for the review and how studies were grouped for the syntheses. | 7-8 |
| Information sources | 6 | Specify all databases, registers, websites, organisations, reference lists and other sources searched or consulted to identify studies. Specify the date when each source was last searched or consulted. | 7-8 |
| Search strategy | 7 | Present the full search strategies for all databases, registers and websites, including any filters and limits used. | 7-8, Supplementary Material S2, S3 |
| Selection process | 8 | Specify the methods used to decide whether a study met the inclusion criteria of the review, including how many reviewers screened each record and each report retrieved, whether they worked independently, and if applicable, details of automation tools used in the process. | 7-8 |
| Data collection process | 9 | Specify the methods used to collect data from reports, including how many reviewers collected data from each report, whether they worked independently, any processes for obtaining or confirming data from study investigators, and if applicable, details of automation tools used in the process. | 9 |
| Data items | 10a | List and define all outcomes for which data were sought. Specify whether all results that were compatible with each outcome domain in each study were sought (e.g. for all measures, time points, analyses), and if not, the methods used to decide which results to collect. | 7 |
|  | 10b | List and define all other variables for which data were sought (e.g. participant and intervention characteristics, funding sources). Describe any assumptions made about any missing or unclear information. | 7 |
| Study risk of bias assessment | 11 | Specify the methods used to assess risk of bias in the included studies, including details of the tool(s) used, how many reviewers assessed each study and whether they worked independently, and if applicable, details of automation tools used in the process. | 9, Supplementary Material S4 |
| Effect measures | 12 | Specify for each outcome the effect measure(s) (e.g. risk ratio, mean difference) used in the synthesis or presentation of results. | 9, Supplementary Material S4 |
| Synthesis methods | 13a | Describe the processes used to decide which studies were eligible for each synthesis (e.g. tabulating the study intervention characteristics and comparing against the planned groups for each synthesis (item #5)). | 9, Supplementary Material S4 |
|  | 13b | Describe any methods required to prepare the data for presentation or synthesis, such as handling of missing summary statistics, or data conversions. | 9, Supplementary Material S4 |
|  | 13c | Describe any methods used to tabulate or visually display results of individual studies and syntheses. | 9, Supplementary Material S4 |
|  | 13d | Describe any methods used to synthesize results and provide a rationale for the choice(s). If meta-analysis was performed, describe the model(s), method(s) to identify the presence and extent of statistical heterogeneity, and software package(s) used. | 9, Supplementary Material S4 |
|  | 13e | Describe any methods used to explore possible causes of heterogeneity among study results (e.g. subgroup analysis, meta-regression). | 9, Supplementary Material S4 |
|  | 13f | Describe any sensitivity analyses conducted to assess robustness of the synthesized results. | N/A |
| Reporting bias assessment | 14 | Describe any methods used to assess risk of bias due to missing results in a synthesis (arising from reporting biases). | 9, Supplementary Material S4 |
| Certainty assessment | 15 | Describe any methods used to assess certainty (or confidence) in the body of evidence for an outcome. | 9, Supplementary Material S4 |
| **RESULTS** | | |  |
| Study selection | 16a | Describe the results of the search and selection process, from the number of records identified in the search to the number of studies included in the review, ideally using a flow diagram. | 10-11 |
|  | 16b | Cite studies that might appear to meet the inclusion criteria, but which were excluded, and explain why they were excluded. | 10-11 |
| Study characteristics | 17 | Cite each included study and present its characteristics. | 10-11 |
| Risk of bias in studies | 18 | Present assessments of risk of bias for each included study. | 9, Supplementary Material S4 |
| Results of individual studies | 19 | For all outcomes, present, for each study: (a) summary statistics for each group (where appropriate) and (b) an effect estimate and its precision (e.g. confidence/credible interval), ideally using structured tables or plots. | 9, Supplementary Material S4 |
| Results of syntheses | 20a | For each synthesis, briefly summarise the characteristics and risk of bias among contributing studies. | 9, Supplementary Material S4 |
|  | 20b | Present results of all statistical syntheses conducted. If meta-analysis was done, present for each the summary estimate and its precision (e.g. confidence/credible interval) and measures of statistical heterogeneity. If comparing groups, describe the direction of the effect. | 9, Supplementary Material S4 |
|  | 20c | Present results of all investigations of possible causes of heterogeneity among study results. | 9, Supplementary Material S4 |
|  | 20d | Present results of all sensitivity analyses conducted to assess the robustness of the synthesized results. | 9, Supplementary Material S4 |
| Reporting biases | 21 | Present assessments of risk of bias due to missing results (arising from reporting biases) for each synthesis assessed. | 9, Supplementary Material S4 |
| Certainty of evidence | 22 | Present assessments of certainty (or confidence) in the body of evidence for each outcome assessed. | 9, Supplementary Material S4 |
| **DISCUSSION** | | |  |
| Discussion | 23a | Provide a general interpretation of the results in the context of other evidence. | 12-14 |
|  | 23b | Discuss any limitations of the evidence included in the review. | 14-15 |
|  | 23c | Discuss any limitations of the review processes used. | 14-15 |
|  | 23d | Discuss implications of the results for practice, policy, and future research. | 15 |
| **OTHER INFORMATION** | | |  |
| Registration and protocol | 24a | Provide registration information for the review, including register name and registration number, or state that the review was not registered. | 6 |
|  | 24b | Indicate where the review protocol can be accessed, or state that a protocol was not prepared. | 6 |
|  | 24c | Describe and explain any amendments to information provided at registration or in the protocol. | N/A |
| Support | 25 | Describe sources of financial or non-financial support for the review, and the role of the funders or sponsors in the review. | 1, Title Page |
| Competing interests | 26 | Declare any competing interests of review authors. | 1, Title Page |
| Availability of data, code and other materials | 27 | Report which of the following are publicly available and where they can be found: template data collection forms; data extracted from included studies; data used for all analyses; analytic code; any other materials used in the review. | 6, 9 Supplementary Material S2-4 |
